# Tracking prodromal Parkinson’s disease: a five-year follow-up of the PARCAS cohort

**DOI:** 10.3389/fneur.2025.1631165

**Published:** 2025-09-12

**Authors:** Kristina Kulcsarova, Petronela Christova, Martina Bekeova, Sona Muranska, Sona Mrazova, Zuzana Mrazova, Barbora Zecova, Filip Faglic, Simona Suvakova, Norbert Lesko, Laura Gombosova, Zuzana Gdovinova, Matej Skorvanek

**Affiliations:** ^1^Department of Neurology, University of Pavol Jozef Safarik, Kosice, Slovakia; ^2^Department of Neurology, University Hospital L. Pasteur, Kosice, Slovakia; ^3^Department of Clinical Neurosciences, University Scientific Park MEDIPARK, University of Pavol Jozef Safarik, Kosice, Slovakia; ^4^2nd Department of Internal Medicine, University of Pavol Jozef Safarik, Kosice, Slovakia; ^5^2nd Department of Internal Medicine, University Hospital L. Pasteur, Kosice, Slovakia

**Keywords:** Parkinson’s disease, prodromal symptoms, MDS prodromal research criteria, alpha-synuclein, synucleinopathies, biomarkers

## Abstract

**Background:**

The updated International Parkinson and Movement Disorders Society (MDS) research criteria for prodromal Parkinson’s disease (pPD) enable pPD probability assessment. In the PARkinson’s disease associated Colonic Alpha-Synuclein biomarker (PARCAS) study, we previously identified 12 possible (7.5%) and 10 probable (6.3%) pPD cases among 160 elderly individuals undergoing colonoscopy at baseline.

**Objective:**

To apply MDS pPD criteria in the PARCAS cohort at five-year follow-up (FU), evaluating pPD detection, longitudinal stability, and conversion rates to Parkinson’s disease (PD) or other neurodegenerative diseases.

**Methods:**

We assessed all risk and prodromal markers except genetic testing; DaTscan and polysomnography (PSG)-confirmed idiopathic REM sleep behavior disorder (iRBD) were available only in a subset of participants. Criteria accuracy was retrospectively evaluated in phenoconverters.

**Results:**

Among 87/160 participants completing FU, 3 possible (3.5%) and 6 probable (7%) pPD cases were detected. Most remained stable in pPD classification (73 negative, 2 possible, 2 probable pPD), while 4 regressed and 5 progressed in their risk category. Two patients converted to PD and one to corticobasal syndrome (CBS). Baseline sensitivity was 0% at 80% probable pPD threshold (rising to 66% at 50% possible pPD threshold) and reached 100% at 80% threshold at FU.

**Conclusion:**

pPD probability showed high agreement between baseline and FU assessments. However, absence of key specific markers (PSG-confirmed iRBD and DaTscan) limited baseline sensitivity, which improved only near phenoconversion as additional prodromal symptoms accumulated. Identification of a prodromal CBS case illustrates the potential for detection of atypical parkinsonisms, even with non-α-synuclein pathology, suggesting limited specificity for PD.

## 1 Introduction

Parkinson’s disease (PD) is the most common α-synucleinopathy and the second most common neurodegenerative disease, with a prodromal phase characterized by a combination of predominantly non-motor symptoms that precede the onset of evident parkinsonism by years (1). In 2015, the International Parkinson and Movement Disorders Society (MDS) research criteria for prodromal PD (pPD) were published, allowing for the calculation of pPD probability based on the presence of risk factors and prodromal markers (2). In 2019, the updated MDS research criteria for pPD refined the numeric values of previously included markers and introduced new subcategories (genetic background and neurogenic orthostatic hypotension), as well as additional risk factors and prodromal markers (3).

Over the last decade, numerous studies have prospectively validated both sets of criteria in different types of target populations, ranging from general elderly community-based studies to various pre-selected, enriched cohorts (mostly high-risk patients with idiopathic REM Sleep Behavior Disorder/iRBD/, hyposmia, carriers of PD-associated genetic mutations such as *GBA1* or *LRRK2*, and other composite cohorts recruited via additional prodromal markers), with diverse sensitivity and specificity outcomes, as reviewed in Kulcsarova and Skorvanek (4).

In the search for a PD biomarker beyond the predominantly clinical diagnosis of pPD, the role of tissue biopsies in assessing the presence of pathological α-synuclein (α-syn) *in vivo* has gained considerable scientific attention in recent years (5). Gastrointestinal tissues have been of particular interest, as α-syn aggregates are detectable as early as the prodromal stage [e.g., (6–9)], and these tissues are readily accessible through routine diagnostic colonoscopies performed in elderly population.

Our previous work aimed to determine the prevalence of pPD among such patients. The original MDS pPD criteria were used for baseline cross-sectional pPD identification in the PARkinson’s disease associated Colonic Alpha-Synuclein biomarker (PARCAS) study cohort (10). Additionally, we reported pathological α-syn accumulation, detected immunohistochemically using the 5G4 antibody, in colonic biopsies of 57% of pPD subjects in a pilot subgroup of the cohort (11).

The aim of the current follow-up study was to apply MDS pPD criteria in the PARCAS cohort, at baseline and at re-examination after five years, to evaluate pPD detection and the rate of conversion to manifest PD or other neurodegenerative diseases.

## 2 Materials and methods

### 2.1 Participants

The PARCAS cohort was prospectively recruited in a single academic center at the University Hospital of L. Pasteur in Kosice, Slovakia, based on the presence of gastrointestinal symptoms such as constipation or at least one other prodromal symptom of PD (hyposmia, questionnaire-based probable iRBD or depression) in elderly patients undergoing diagnostic colonoscopies (10). Exclusion criteria included manifest parkinsonism or presence of any other neurodegenerative disease, age below 40 years, active colonic cancer or inflammatory bowel disease, or expected survival less than 3 years based on other comorbidities. Baseline examinations were performed between 2014 and 2018, with 160 subjects enrolled in. Follow-up (FU) examinations were carried out approximately 5 years later, between 2018 and 2024. The study was performed according to the Declaration of Helsinki of 1975; it was approved by the local ethics committee and all participants signed written informed consent prior to enrollment.

### 2.2 Calculation of prodromal Parkinson’s disease probability based on the MDS research criteria

Since the baseline examinations (starting in 2014) were initiated before the publication of both the original (2015) (2) and updated (2019) (3) MDS pPD research criteria, only a limited subset of risk factors and prodromal markers—those already recognized in the field—were included in the original study protocol. Although the selection was made prior to the release of the original criteria, the list of markers assessed at baseline corresponded closely to that later defined in the 2015 criteria, except for genetic testing and DaTscan. Some of the markers were assessed using simplified methods (e.g., binary questionnaire items rather than standardized rating scales; polysomnography (PSG) for iRBD confirmation was performed only in a subset of the cohort). At FU, the assessment protocol was expanded and updated in accordance with the updated MDS criteria, excluding only genetic testing; DaTscan was performed in a selected subset of high-risk individuals. The FU protocol was also harmonized with our concurrent prospective Parkinson’s Disease BIOMarker (PDBIOM) study evaluating individuals with iRBD, as described previously (10, 12).

To ensure consistency and enable comparison between diagnostic frameworks, we performed parallel calculations of pPD probability using both the original and updated MDS research criteria. For analysis based on the original criteria, only the limited list of markers defined in that version was used at both timepoints. Positive/negative likelihood ratios (LRs) were applied as originally specified, regardless of whether additional data were available at FU. In contrast, analyses based on the updated criteria included all available markers at each timepoint, applying the revised LRs. At baseline, only the originally collected markers were used (without retrospective supplementation), but were evaluated according to the updated 2019 LRs where applicable. At FU, a broader range of markers was assessed and incorporated, consistent with the 2019 criteria.

Total LRs for each individual were calculated by multiplying the respective LRs of all included markers. Final post-test pPD probability was then derived using the total LR and age-related pretest probability, as defined in the MDS pPD research criteria. Participants were subsequently classified into three categories: probable pPD (≥80% probability), possible pPD (50–79%), and not fulfilling pPD criteria (<50%).

Detailed information on diagnostic tools and cut-off criteria for LRs assignment for all risk and prodromal markers is provided in Supplementary Materials (Data Sheet 1); a visual summary of marker availability and assessment methods at both timepoints is provided in Supplementary Table 1.

### 2.3 Statistical analyses

Statistical analyses were performed using the statistical software program PASW SPSS version 31.0 for Windows (SPSS Inc., Chicago IL). Descriptive statistical methods were used to summarize sociodemographic and clinical characteristics and to determine the prevalence of pPD based on the MDS research criteria. To compare the detection rates of possible and probable pPD between baseline and FU, the Stuart-Maxwell test (marginal homogeneity test) was used to evaluate categorical status changes, and the Wilcoxon signed-rank test was used to assess changes in individual pPD probability scores. To address potential attrition bias, multiple imputation using the fully conditional specification (FCS) method was conducted to estimate missing follow-up data. Baseline age, sex, education, risk factors/prodromal markers and pPD probability scores were used as predictors. Variables with excessive missingness (e.g., >90%) were excluded from the imputation model to improve model stability and retain more cases. The sex-specific variable erectile dysfunction, available only in men, was omitted from dropout modeling to avoid structural missingness. Ten imputed datasets were generated and pooled using Rubin’s rules. Binary logistic regression was then used to model dropout status based on baseline characteristics. Imputed datasets were also used in sensitivity analyses (Wilcoxon signed-rank test and marginal homogeneity test) to assess longitudinal changes in pPD probability and classification while accounting for missing FU data. Given the exploratory nature of some analyses and the limited sample size, no formal correction for multiple comparisons was applied.

## 3 Results

To enable evaluation of classification differences between the two versions of the MDS pPD research criteria, we applied both the original (2015) and updated (2019) diagnostic frameworks in parallel. However, as the original criteria are now considered outdated, detailed results based on that framework are presented only in the Supplementary Results (Data Sheet 2) (including Supplementary Figure 1 and Supplementary Tables 5, 6) and are not emphasized in the main text. The following results focus on analyses based on the updated 2019 MDS pPD criteria.

### 3.1 Prodromal Parkinson’s disease identification (baseline and follow-up)

At baseline, 160 subjects with sufficient data for pPD probability calculation were included in the PARCAS cohort (64 males—40%, 96 females—60%, mean age 62.36 ± 9.16 years). Updated MDS pPD criteria detected 12 possible (7.5%) and 10 probable (6.3%) pPD cases.

After approximately 5 years, 87 participants (54.4%) completed follow-up evaluations (35 males—40.2%, 52 females—59.8%, mean age 67.18 ± 8.58 y., mean time to FU: 5.46 ± 1.24 y.). This FU subgroup of the cohort included 9 possible (10.3%) and 3 probable (3.4%) pPD cases at baseline. At FU, one patient had already phenoconverted to manifest neurodegenerative disease and was excluded from further pPD assessment. For the remaining 86 subjects, 3 possible (3.5%) and 6 probable (7%) pPD cases were detected, while 77 subjects (89.5%) still did not meet pPD criteria.

To assess potential attrition bias, dropout was modeled using binary logistic regression based on baseline characteristics in multiple imputed datasets (*n* = 10). In the original dataset, 60 cases (37.5%) had at least some missing baseline predictor data and would have been excluded from complete-case regression; thus, multiple imputations were necessary to preserve statistical power and avoid potential bias. The pooled model included demographic variables, risk factors, prodromal markers, and baseline pPD probability. Results revealed that only lower education was significantly associated with dropout (B = −0.125, SE = 0.060, *p* = 0.037, OR = 0.882, 95% CI: 0.784–0.993), while no other predictors—including baseline pPD probability—showed significant effects. Model fit statistics (Nagelkerke R^2^ ranging from 0.201 to 0.249, average 0.227) indicate modest explanatory power, and pooled variance metrics (relative efficiency ≥ 0.96) reflect robust efficiency across imputations. These findings support the validity of the overall results despite the relatively high dropout rate.

**Figure 1:**
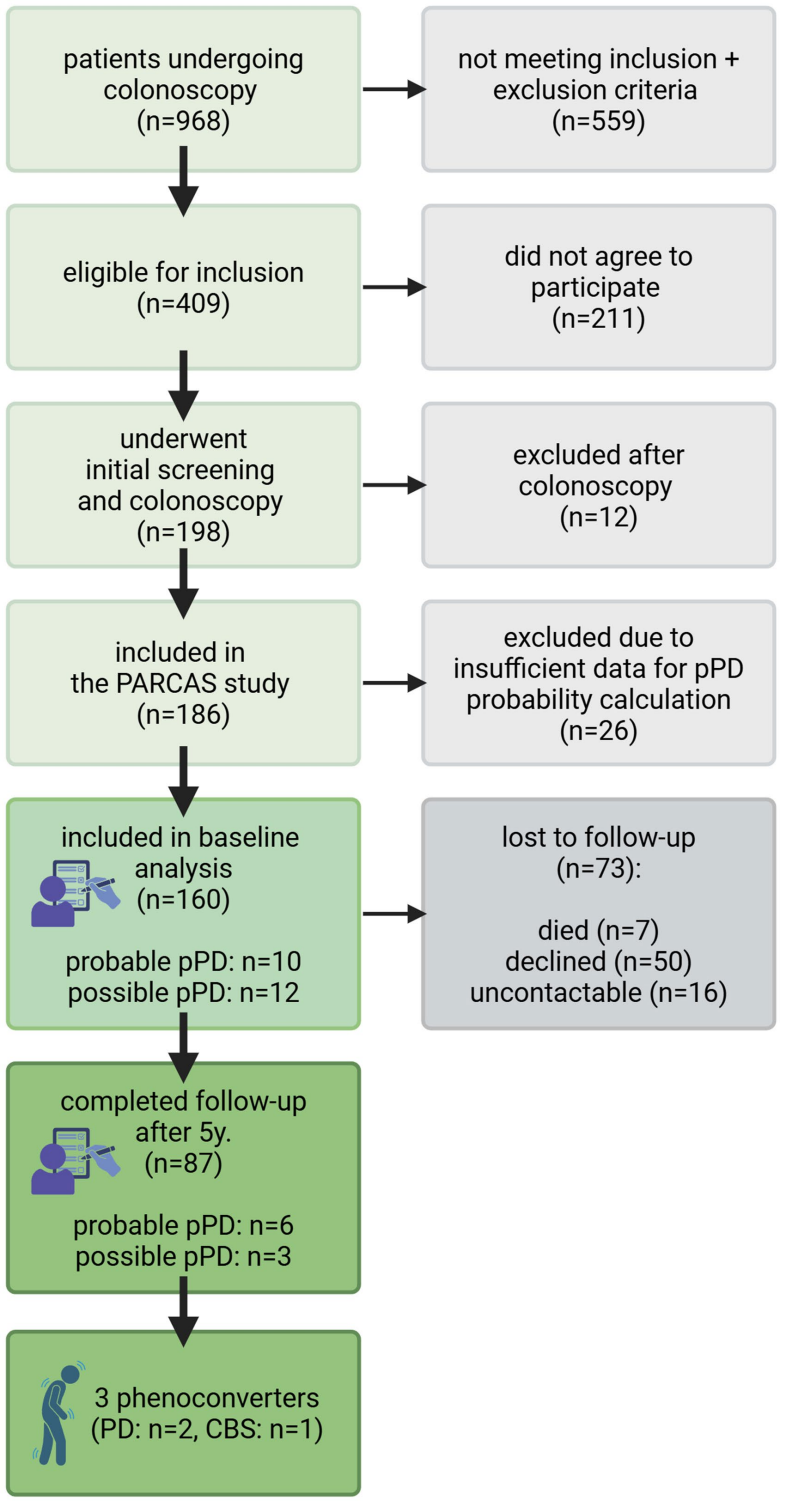
PARCAS cohort: recruitment, baseline analysis (2014–2018) and follow-up examinations (2018–2024). Numbers of pPD patients at baseline and follow-up based on the updated MDS pPD research criteria. CBS: corticobasal syndrome; PARCAS: PARkinson’s disease associated Colonic Alpha-Synuclein biomarker study; PD: Parkinson’s disease; pPD: prodromal Parkinson’s disease; y: years.

The recruitment process, baseline analysis, and FU examinations are summarized in Figure 1, while basic sociodemographic data and prevalence of risk factors and prodromal markers at baseline and FU are detailed in Table 1. Complete regression results for all predictors are provided in Supplementary Table 2.

**Table 1:** Characteristics of the PARCAS cohort at baseline and follow-up and prevalence of risk factors and prodromal markers based on the MDS pPD research criteria.

Cohort	Entire PARCAS cohort	FU subgroup of the PARCAS cohort	FU subgroup of the PARCAS cohort
Examination	Baseline	Baseline	FU
Basic sociodemographic data			
*N* of participants	160	87	87
Age (y., mean ± SD)	62,36 ± 9.16	61.85 ± 8.78	67.18 ± 8.58
Gender (*n*, %)	M: 64 (40%), F: 96 (60%)	M: 35 (40.2%), F: 52 (59.8%)	M: 35 (40.2%), F: 52 (59.8%)
Years of education (mean ± SD)	13.41 ± 3.49	14.1 ± 3.32	14.42 ± 3.03
Prevalence of risk factors			
Pesticide exposure (*n*, %)*	39/150 (26%)	20/82 (24.4%)	4/83 (4.8%)
Solvent exposure (*n*, %)*	9/145 (6.2%)	3/79 (3.8%)	7/83 (8.4%)
Non-use of caffeine (*n*, %)*	51/149 (34.2%)	24/82 (29.3%)	14/83 (16.9%)
Smoking status			
Never (*n*, %)*	96/147 (65.3%)	51/80 (63.8%)	58/86 (67.4%)
Former (*n*, %)*	37/147 (25.2%)	20/80 (25%)	22/86 (25.6%)
Current (*n*, %)*	14/147 (9.5%)	9/80 (11.3%)	6/86 (7.0%)
Positive family history of PD (*n*, %)*	24/155 (15.5%)	14/85 (16.5%)	18/87 (20.7%)
SN hyperechogenicity			
≥0.25 cm^2^ (*n*, %)*	14/125 (11.2%)	9/77 (11.7%)	9/81 (11.1%)
≥0.20 and <0.25 cm^2^ (*n*, %)*	8/125 (6.4%)	6/77 (7.8%)	7/81 (8.6%)
<0.20 cm^2^ (*n*, %)*	85/125 (68.0%)	55/77 (71.4%)	55/81 (67.9%)
No temporal window (*n*, %)*	18/125 (14.4%)	7/77 (9.1%)	10/81 (12.3%)
DM type 2 (*n*, %)*	–	–	13/71 (18.3%)
Physical inactivity (*n*, %)*	–	–	19/53 (35.8%)
Low plasma urate in men (*n*, %)*	–	–	8/31 M (25.8%)
Prevalence of prodromal markers			
PSG-confirmed iRBD (*n*, %)*	3/5 (60%)	1/2 (50%)	1/1 (100%)
RBDSQ ≥5 points (*n*, %)*	18/145 (12.4%)	9/83 (10.8%)	6/87 (6.9%)
Abnormal DaTscan (*n*, %)*	–	–	2/3 (66.7%)
Subthreshold parkinsonism (*n*, %)*	63/158 (39.9%)	39/87 (44.8%)	15/87 (17.2%)
Hyposmia (*n*, %)*	52/157 (33.1%)	26/86 (30.2%)	19/87 (21.8%)
Constipation (*n*, %)*	64/155 (41.3%)	36/86 (41.9%)	6/87 (6.9%)
Excessive daytime somnolence (*n*, %)*	36/151 (23.8%)	18/84 (21.4%)	7/85 (8.2%)
Symptomatic hypotension (*n*, %)*	10/151 (6.6%)	3/84 (3.6%)	10/87 (11.5%)
Erectile dysfunction (*n*, %)*	9/54 M (16.7%)	6/32 M (18.8%)	8/32 M (25%)
Urinary dysfunction (*n*, %)*	25/153 (16.3%)	14/85 (16.5%)	21/87 (24.1%)
Depression (*n*, %)*	26/156 (16.7%)	16/86 (18.6%)	20/87 (23.5%)
Global cognitive deficit (*n*, %)*	–	–	52/87 (59.8%)
pPD identification based on the MDS pPD research criteria			
**Original MDS criteria (2015)**			
Probable pPD (*n*, %)	9/160 (5.6%)	2/87 (2.3%)	3/86 (3.5%)
Possible pPD (*n*, %)	9/160 (5.6%)	8/87 (9.2%)	4/86 (4.7%)
**Updated MDS criteria (2019)**			
Probable pPD (*n*, %)	10/160 (6.3%)	3/87 (3.4%)	6/86 (7%)
Possible pPD (*n*, %)	12/160 (7.5%)	9/87 (10.3%)	3/86 (3.5%)

* All results are given in absolute numbers (*n* positive/*n* tested) and valid percentages (after removing missing data). DaT: dopamine transporter; DM: diabetes mellitus; FU: follow-up; M/F: male/female; MDS: International Parkinson and Movement Disorders Society; n: number; PARCAS: PARkinson’s disease associated Colonic Alpha-Synuclein biomarker study; PD: Parkinson’s disease; PSG: polysomnography; RBD: REM Sleep Behavior Disorder; RBDSQ: RBD Screening Questionnaire; pPD: prodromal Parkinson’s disease; SD: standard deviation; SN: substantia nigra; y: years.

### 3.2 Evolution of prodromal Parkinson’s disease probability in individual participants (baseline vs. follow-up)

At FU, 77 participants (89.5%) remained in the same pPD category as at baseline (73 still negative, 2 still possible, 2 still probable pPD). 9 participants (10.5%) experienced a categorical shift—4 individuals (4.7%) no longer met pPD criteria (3 previously possible and 1 previously probable pPD cases were reclassified as negative), while 5 subjects (5.8%) progressed to a higher-risk category (2 newly identified cases: 1 possible, 1 probable pPD; and 3 progressed from possible to probable pPD). Regarding changes in continuous pPD probability scores, 48 subjects (55.8%) experienced a decrease, 37 (43.0%) an increase, and 1 (1.2%) showed no change over time.

The complete-case Wilcoxon signed-rank test indicated a non-significant trend toward decreasing pPD probability from baseline to FU (Z = −1.461, *p* = 0.144, small estimated effect size: r = 0.158). In contrast, analyses across the imputed datasets revealed a slight predominance of increasing over decreasing ranks, suggesting a mild upward trend in pPD probability; nevertheless, these changes also failed to reach statistical significance (average Z = −1.179, average *p* = 0.243, small estimated effect size: average r = 0.096).

For categorical shifts, the marginal homogeneity test (Stuart–Maxwell test) in the complete-case sample did not reveal a significant change in pPD status (Z = −0.258, *p* = 0.796, negligible estimated effect size: r = 0.028). However, when FU pPD status was rederived from imputed probability scores across the 10 datasets, the marginal homogeneity test revealed a statistically significant shift toward lower-risk prodromal categories (average Z = 2.359, average *p* = 0.018, small estimated effect size: average r = 0.187).

**Figure 2:**
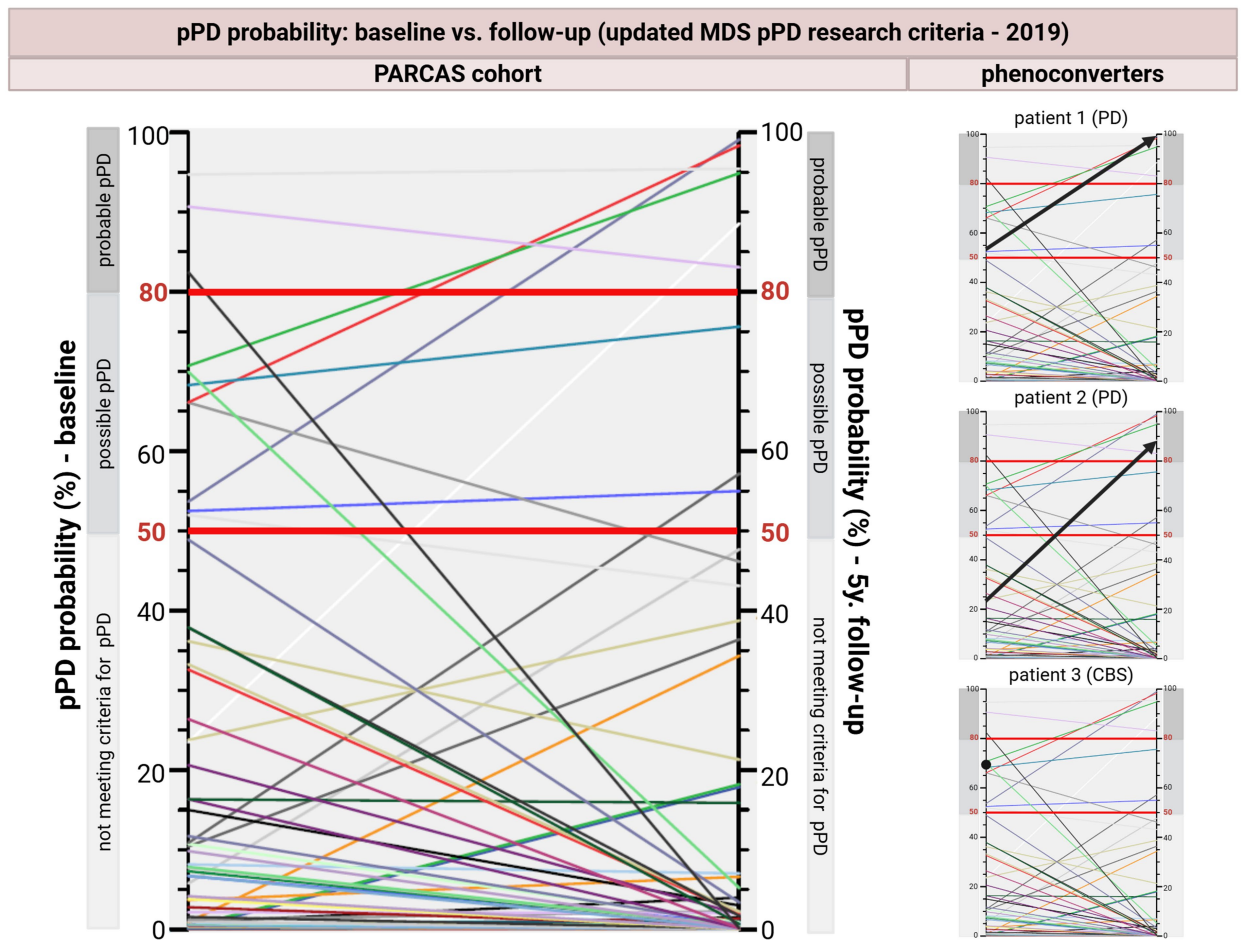
Evolution of prodromal Parkinson’s disease probability scores as defined by the updated MDS pPD research criteria—from baseline to follow-up after 5 years. Left side: summary graph for the entire PARCAS cohort; right side: individual phenoconverted patients highlighted; red lines—50 and 80% probability cut-offs for possible and probable pPD, respectively; individual narrow color lines representing individual participants and their % pPD probability change from baseline to follow-up; individual bold black arrows/dot representing phenoconvertes. CBS: corticobasal syndrome; pPD: prodromal Parkinson’s disease; y: years.

pPD probability changes from baseline to FU in the complete-case sample are presented in Figure 2—for both the entire cohort and individual phenoconverters; complete Wilcoxon signed-ranks test results for changes in pPD probability scores are provided in Supplementary Table 3; complete marginal homogeneity test results for changes in pPD category are provided in Supplementary Table 4.

### 3.3 Phenoconverters

To date, 3/87 subjects (3.4%) have phenoconverted to manifest neurodegenerative disease: 2 patients developed PD (2.3%) and 1 patient converted to corticobasal syndrome (CBS; 1.1%). Detailed clinical phenotypes and MDS pPD scores are outlined below.

Patient 1 (KE-1049) had several risk factors and prodromal markers already at baseline—pesticide exposure, nonsmoking, subthreshold parkinsonism, hyposmia, and constipation (pPD probability: original MDS criteria—42.4%, updated MDS criteria—53.7%, meeting possible pPD category). At FU, new markers emerged—solvent exposure, physical inactivity, abnormal DaTscan, urinary dysfunction, and global cognitive deficit (pPD probability: original criteria—96.9%, updated criteria—99.1%; probable pPD in both cases). Definite phenoconversion to overt clinically manifest PD occurred approximately a year post-FU.

Patient 2 (KE-2019) presented with male sex, nonsmoking, nonuse of caffeine, hyperechogenicity of the substantia nigra, and subthreshold parkinsonism at baseline, but did not meet pPD criteria (pPD probability: original criteria—31.5%, updated criteria—23.5%). At FU, type 2 diabetes mellitus, erectile dysfunction and cognitive deficit expanded the list, further increasing pPD probability (original criteria—64.7%—possible pPD, updated criteria—88.6%—probable pPD). Phenoconversion to manifest PD followed within a year after FU. Although this patient had undergone DaTscan prior to baseline (outside of the research protocol, as part of routine differential diagnostic workup for long-standing comorbid essential tremor), the result showed only minimal, nonspecific asymmetry, far from the clear abnormality typically associated with PD. Based on this, we would have assigned a borderline likelihood ratio (LR = 1), which would not have influenced the final pPD probability. As he was the only subject in the cohort with an available DaTscan result at baseline, and given its equivocal nature, this finding was excluded from the baseline probability calculation. At FU, the patient declined a repeat DaTscan. However, after clinical phenoconversion one year later, a DaTscan was performed and showed a clearly abnormal result, consistent with a diagnosis of PD.

Patient 3 (KE-2052) had a combination of male sex, nonsmoking, hyperechogenicity of the substantia nigra, hyposmia, constipation and subthreshold parkinsonism at baseline, leading to possible pPD status (pPD probability: original criteria—60.2%, updated criteria—69.5%). At FU, there was evident parkinsonism and pyramidal lesion with significant asymmetry (right-sided dominance on extremities), dystonia, spasticity, alien limb phenomenon, motor apraxia, cortical sensory deficit, and executive dysfunction, accompanied by an abnormal DaTscan (more pronounced on the left side). Due to already manifest neurodegenerative disease, pPD probability was not recalculated at FU. Based on the described clinical manifestation, diagnosis of CBS was made, as published previously (4).

### 3.4 Retrospective sensitivity of the MDS pPD criteria in phenoconverters

Sensitivity was calculated based solely on the phenoconverters in the complete-case sample (3/87), as imputing phenoconversion status is not methodologically appropriate. Other diagnostic metrics—such as specificity, predictive values, or accuracy—were not estimated, given the inherent uncertainty in the long-term diagnostic trajectory of non-converting participants. Individuals who have not yet developed PD may still do so in the future, including those currently classified as negative or possible/probable pPD. As such, true-negative status cannot be confirmed at this stage of FU, and any estimates of specificity or predictive values would be premature and potentially misleading.

Although one of the three phenoconverted participants was ultimately diagnosed with CBS, this case was included in the sensitivity analysis as a true positive. While CBS is not the intended diagnostic outcome of the MDS pPD criteria, it is a neurodegenerative parkinsonian disorder, and the subject fulfilled multiple prodromal features consistent with pPD. Excluding this case would underestimate the real-world sensitivity of the criteria, particularly given their current limitations in distinguishing PD from atypical parkinsonisms during the prodromal phase.

Among the three participants who phenoconverted during FU, the performance of the MDS pPD criteria at baseline was mixed. One individual was not identified by either version of the criteria, one was classified as possible pPD only by the updated version, and one met the possible pPD threshold under both the original and updated criteria. Consequently, when applying the 80% probability threshold for probable pPD, baseline sensitivity was 0% for both sets of criteria. Lowering the threshold to 50% for possible pPD improved sensitivity to 33% for the original criteria and 66% for the updated criteria.

At FU, approximately one year before diagnosis, both eventual PD cases were correctly identified as probable pPD using the updated criteria (sensitivity 100% at the 80% threshold). Under the original criteria, one case met the probable pPD threshold, while the second reached only the possible pPD level (sensitivity 50% at the 80% threshold; 100% at the 50% threshold).

## 4 Discussion

The MDS pPD research criteria represent a robust and widely accepted framework for early Parkinson’s disease detection. However, their diagnostic performance and practical applicability are influenced by cohort composition, variability in prodromal marker assessment, and the absence of confirmatory biomarkers. These aspects—including sensitivity, specificity, methodological challenges, and future directions—are discussed in detail below.

### 4.1 Characteristics and longitudinal dynamics of the PARCAS cohort

Our study employed a pragmatic recruitment approach, leveraging patients undergoing colonoscopy to enable access to colonic biopsies for early biomarker exploration. While this design may raise concerns about overrepresentation of gastrointestinal symptoms, participants were included based on the presence of at least one prodromal or risk marker (e.g., constipation, hyposmia, probable iRBD, depression), not strictly on gastrointestinal complaints. Colonoscopy referrals included both symptomatic and routine preventive indications, and only 41% of the cohort reported constipation at baseline. Although this inclusion strategy resulted in a mildly enriched sample, the baseline detected prevalence of pPD remained modest (probable pPD: 6.3%; possible pPD: 7.5%). Most participants were classified as not meeting pPD criteria at either timepoint, and the overall pPD probability distribution more closely resembled that of general aging populations than of highly enriched cohorts such as iRBD samples. This supports the generalizability of our findings beyond gastrointestinal or high-risk clinical settings.

At FU, pPD probability scores demonstrated overall longitudinal stability. While 10.5% of participants experienced categorical changes in pPD classification, the vast majority (89.5%) remained in the same diagnostic category as at baseline. Continuous probability scores showed a balance between upward and downward shifts, with no significant overall trend. Interestingly, imputation-based analyses revealed a statistically significant shift toward lower-risk categories, despite a mild average increase in continuous probability. This seemingly paradoxical result may reflect threshold effects near diagnostic cutoffs, where small fluctuations in input variables can disproportionately affect category assignment due to the non-linear nature of the probability algorithm. In addition, more comprehensive FU assessments or regression to the mean introduced by multiple imputation may have contributed to this pattern. Together, these findings highlight both the robustness of pPD probability as a longitudinal measure and the interpretive complexity of categorical changes in prodromal classification.

Dropout modeling further supported the validity of FU findings. Despite a relatively high attrition rate—45.6% overall and 70% within the baseline probable pPD subgroup (at least partially attributable to their higher mean age at baseline: 70.4 ± 6.48 years vs. 62.36 ± 9.16 years in the full cohort, potentially linked to increased comorbidities and mortality: 3/10 in the probable pPD group vs. 4/150 in the remainder of the cohort)—missingness was not significantly associated with baseline pPD probability or other clinical markers, with the exception of lower education. These findings suggest that attrition bias likely had only a limited impact on the observed evolution of pPD classification over time.

### 4.2 Sensitivity of the MDS pPD criteria

In our study, the updated criteria showed modestly improved sensitivity compared to the original version, with a further increase over time due to higher pPD probability scores at FU driven by accumulating prodromal markers. However, these observations must be interpreted with caution given the small number of converters (*n* = 3), which limits the robustness of any sensitivity estimates. Similar trends have been observed in other non-phenoconverted subjects, though further longitudinal follow-up is needed to assess eventual future conversion. Nonetheless, the low sensitivity at baseline is in line with expectations for a cohort with heterogenous composition, lacking high-LR + markers such as PSG-confirmed iRBD or DaTscan for all participants. This is consistent with findings from other population-based cohorts, where reliance on lower-LR clinical markers alone often proves insufficient for early detection, with sensitivity rising only shortly before phenoconversion.

Comparable tendencies have been reported in other studies. For example, iRBD cohorts have shown substantially higher sensitivity [e.g., original criteria at 80% threshold: 66.7% at baseline and 100% at last visit before conversion (13), or 80% sensitivity at 80% threshold and 100% sensitivity at 50% threshold (14)]. However, general elderly population cohorts, where high-LR + markers are absent, report much lower sensitivity [e.g., original criteria—HELIAD cohort: 0% at 80% threshold, 4.5% at 50% threshold (15), PRIPS cohort: 0% at 80, 14% at 50% (15, 16), Bruneck cohort at 10y. FU: 35% at 80, 60% at 50% (17); updated criteria—HELIAD cohort: 0% at 80, 4.5% at 50% (15); Bruneck cohort at 10y. FU: 40% at 80, 65% at 50% threshold (18)]. Recent largest population-based Lifelines cohort, despite including 160 PD-converters, found that none of them exceeded 80% or even 50% threshold retrospectively using a limited set of self-reported or questionnaire-based markers alone (19). Even in a *de novo* PD cohort, the original criteria had only 21.8% sensitivity at 80% threshold (14). Thus, while the MDS criteria perform well in high-risk cohorts, their widespread application in low-risk populations is much less effective (4).

These comparisons highlight the importance of high-LR + markers for increasing sensitivity. At the same time, they reflect a central limitation of community-based studies: the ethical and logistical barriers to systematically implementing comprehensive diagnostic protocols, including expensive or invasive diagnostic tests. In our study, some high-LR + tests such as PSG or genetic testing were not applied systematically due to feasibility constraints; DaTscan was selectively offered only to high-risk individuals (partly due to ethical considerations around exposing healthy participants to ionizing radiation without clinical justification) and some individuals declined despite high predicted risk. These limitations mirror the broader challenge of balancing scientific rigor with participant safety and acceptability in large-scale screening efforts.

Therefore, while our findings suggest that the updated criteria may help identify emerging prodromal features over time, the limited number of converters and the reliance on lower-LR clinical markers constrain sensitivity—underscoring the need for biomarker-enriched approaches in future studies and follow-ups.

### 4.3 Specificity of the MDS pPD criteria and differential diagnostics

Two key specificity challenges emerged in our cohort. First, false positivity: identification of subjects as pPD based on relatively common and nonspecific symptoms such as constipation, depression, or urinary dysfunction that may result from unrelated conditions. A recent study screening for several pPD symptoms (hyposmia, cognitive impairment, patient-reported constipation, possible iRBD, depression, and anxiety) in a late middle-aged population found 3–13% prevalence for individual prodromal markers, with 11% of subjects reporting two or more (20). Moreover, some symptoms can eventually regress over time (as seen in our 4 subjects initially identified as pPD and later re-classified as negative due to a reduced number of previously reported symptoms). Similar pattern was reported in the HELIAD cohort, where several subjects initially classified as probable pPD decreased their risk scores at FU even below 30% threshold (15).

The second aspect relates to the differentiation between PD and other neurodegenerative parkinsonisms. Among the three converters in our cohort, one participant—classified as possible pPD at baseline—was later diagnosed clinically with CBS. While this might illustrate a limitation of the criteria in distinguishing PD from atypical forms of parkinsonism, we emphasize that this is a single case and should be interpreted as a preliminary observation rather than definitive evidence of limited specificity. We have no pathological confirmation of the underlying neurodegenerative process in this case, and the participant is still alive. Although CBS is most commonly associated with 4R tauopathies such as corticobasal degeneration (CBD), its neuropathological spectrum is heterogeneous. In addition to CBD, other underlying pathologies such as progressive supranuclear palsy, Alzheimer’s disease (AD), TDP-43 proteinopathy, fused in sarcoma (FUS) proteinopathy, prion disease, cerebrovascular pathology, and in rare instances even synucleinopathies have been reported (21). Notably, AD pathology—frequently found in CBS—has increasingly been shown to include α-syn co-pathology in a substantial proportion of cases (22, 23). This raises the possibility that a diagnosis of CBS does not necessarily preclude the presence of underlying synucleinopathy. Indeed, the fact that this individual met the MDS pPD criteria at baseline may even suggest an underlying α-syn-related process, diminishing its value as a clear example of cross-specificity failure. Thus, rather than being viewed as a demonstration of poor specificity, this case highlights the biological and clinical complexity of parkinsonian disorders and the potential overlap of prodromal features across different underlying pathologies.

Beyond this single case, challenges in differential diagnosis are well known, particularly in cohorts enriched for iRBD or autonomic dysfunction. While the specificity of the criteria versus healthy controls surpasses 90% (4), specificity against other synucleinopathies is lower. iRBD is a core prodromal feature not only of PD, but also of dementia with Lewy bodies (DLB), and multiple system atrophy (MSA). According to a recent multicenter analysis, 58.6% of iRBD patients converted to parkinsonism-first phenotype (including both PD, 53.6%, and MSA, 5%), while 41.4% developed DLB (24). Accordingly, DLB and MSA are often considered valid phenoconversion outcomes equivalent to PD in some iRBD-based cohorts (13–15). As reflected in current research criteria for pPD (2, 3), prodromal DLB (pDLB) (25) and prodromal MSA (pMSA) (26), there is a significant overlap of prodromal phenotypes, until the trajectories finally diverge in later stages. For example, an iRBD patient with additional features such as autonomic dysfunction or mild cognitive impairment may simultaneously meet the criteria for several prodromal synucleinopathies. This anticipated overlap was elegantly illustrated in a recent cross-sectional study applying all three sets of criteria in an iRBD cohort: 96.4% patients met at least one prodromal diagnosis, and 56% met the criteria for more than one definition (32.7% pMSA&pPD, 10.9% pDLB&pPD, and 12.7% all three) (27). This insufficient specificity to differentiate between prodromal synucleinopathies —due to their shared core phenotype —is not a surprise from a research perspective, given that each set of criteria was developed independently to maximize detection sensitivity for each entity. However, this limits their utility for prodromal differential diagnosis or individualized prognostic counseling in clinical setting (27, 28).

In the context of clinical trials, particularly those targeting α-syn, the nonspecific inclusion of individuals with overlapping or atypical pathologies remains a critical concern. If MDS pPD probability scores alone were used for trial selection, cases with underlying non-synucleinopathies might inadvertently be enrolled, potentially diluting treatment effects. This underscores the need for future refinement of prodromal criteria, ideally supported by molecular biomarkers capable of identifying the underlying pathology with greater precision.

### 4.4 Challenges of the clinically based MDS pPD criteria and prodromal markers assessment

While the MDS pPD criteria precisely define the list of risk and prodromal markers along with their respective LRs, they provide less explicit guidance on the recommended diagnostic tools for their identification. Some markers, like smoking history or caffeine consumption, have clear cut-offs, making it easy to incorporate into screening questionnaires. However, others, such as excessive daytime somnolence, symptomatic hypotension, erectile/urinary dysfunction, or depression, lack uniformly defined criteria (2, 3). As a result, different studies applying MDS pPD criteria use a variety of questionnaires and clinical tests, leading to inconsistencies. The criteria attempt to mitigate this by assigning different LRs to the same markers based on the diagnostic method used. For instance, iRBD has an LR of 130 when confirmed by PSG but only 2.8 when diagnosed via questionnaire; a similar approach is used for subthreshold parkinsonism and (neurogenic) orthostatic hypotension. While these stratified LRs account for methodological differences, some prodromal symptoms lack comparable categorization. Yet, variability in screening tools influences detection rates, even for the less significant markers, as seen in our study, where different diagnostic protocols between baseline and FU led to unexpected variations in marker prevalence. Baseline examinations followed a simpler diagnostic approach, whereas FU assessments incorporated more detailed and standardized scales. This shift may partially explain the observed decrease in some markers over time and the overall decline in pPD probability in most participants. For example, stricter cut-off values at FU (based on standardized assessments rather than self-reported data) may have led to lower positivity rates for markers such as constipation.

The lack of a standardized diagnostic protocol allows for notable variability in the diagnostic tools chosen by different research groups to screen for prodromal symptoms, which in turn can impact the overall performance of the MDS criteria. Given the relatively high prevalence of various prodromal markers in the general population (20), it is crucial to establish appropriate cut-off scores to balance sensitivity and specificity in their detection. Moreover, using scales designed for assessing clinically evident symptoms in manifest disease to track prodromal symptoms, particularly motor ones, may be problematic.

This challenge is particularly evident for subthreshold parkinsonism—one of the most influential drivers of the total LR (LR + = 9.6)—defined as a score >6 in the MDS-Unified Parkinson’s Disease Rating Scale (MDS-UPDRS)-III (excluding postural and action tremor) or >3 in the original UPDRS-III (excluding action tremor), as per the MDS research criteria. However, the MDS-UPDRS-III was developed to quantify motor symptoms severity in manifest PD, not for screening in asymptomatic population. The “rate-as-you-see” approach may overestimate scores in individuals without PD but with other comorbidities affecting movements (29). Subthreshold parkinsonism is a late prodromal motor sign, typically appearing within last years before phenoconversion (30, 31). In contrast, mild parkinsonian signs (MPS), which may overlap with the subthreshold parkinsonism construct, are nonspecific motor abnormalities that become more prevalent with age and occur in a substantial portion of PD-free individuals — up to 14.9% of those aged 65–75 and 52.4% of those over 85 (32), far exceeding corresponding age-related prevalence of PD (1% at 70 years, 4% at 85 years) (33). MPS can stem from various conditions, including vascular risk factors, type 2 diabetes, dementia, arthritis, orthopedic diseases, essential tremor and others (34). Although the MDS criteria caution against score overestimation in cases of unequivocal confounders (e.g., bradykinesia misattributed to arthritis or stooped posture due to osteoporotic kyphosis), individuals with MPS due to more subtle or mixed comorbidities may still quite easily exceed the 6-point threshold on the MDS-UPDRS-III, largely due to less specific items (e.g., posture, gait, or global spontaneity of movements). Moreover, the absence of a borderline zone means that a single-point difference in the MDS-UPDRS-III (≤6 vs. >6) can lead to a dramatic shift in LR attribution (from a negative LR of 0.55 to a positive LR of 9.6), resulting in an almost 20-fold difference in the total LR and final pPD probability.

In our cohort, subthreshold parkinsonism was identified in 39.9% of participants at baseline, raising concerns about potential overestimation. Although the same diagnostic criteria and cut-offs were applied consistently at both timepoints, several factors may have contributed to the observed decrease in prevalence to 17.2% at FU. These may include both the natural course of motor abnormalities—such as the reported possibility of MPS regressing over time (particularly in younger individuals) (35), as well as rater-related variability. Across the study period (2014–2024), multiple clinical raters were involved; most were not movement disorders specialists, although all had completed official MDS-UPDRS training. Inter-rater variability, combined with evolving clinical judgment, may have influenced the scoring. Initial assessments may have been more permissive, while later evaluations—possibly shaped by growing experience and awareness of overestimation risk—may have been more conservative.

To overcome these limitations and the potential bias associated with the subjective nature of motor examination, the future likely lies in objective motor assessment. Rapid advances in digital technology enable the detection of subtle motor deficits and their progression over time, with promising markers extracted from voice, arm swing, gait, balance, and breathing (36).

Differences in study protocols across prodromal cohorts contribute to heterogeneity in reported results, highlighting the need for standardized clinical assessment to harmonize pPD detection and improve comparability between studies. However, an even more fundamental issue is the lack of formal guidelines on the use of existing clinical scales for prodromal symptoms screening and the absence of a dedicated scale tailored for this purpose.

### 4.5 Future directions: biomarker-based criteria

These challenges open a discussion on sustainability, purpose and context for the best usability of the current MDS pPD research criteria. It is not questioned that they represent the most complex approach for pPD identification, and their specificity exceeds 90% in many publications. On the other hand, as their application is logistically demanding and time consuming and their outcome is a measure of pPD probability, not a confirmed pPD diagnosis, their use is currently for practical and ethical reasons still limited to research context. The sensitivity of the criteria is high in pre-selected high-risk cohorts (such as iRBD) but decreases markedly in lower-risk populations or non-enriched cohorts. Therefore, their use as a pPD screening tool in the general population is not feasible and recommended. While prodromal CBS identification has not been reported outside of our cohort and does not pose a common problem, it illustrates the possibility of pPD identification in patients that later develop atypical parkinsonism (even other than synucleinopathy-based), which could be a practical problem in α-syn-targeting clinical trials, unless α-syn biomarkers are required for inclusion.

Given these limitations, there is an ongoing shift toward biomarker-based PD diagnostics—a way forward seems to be paved by the two sets of recently published biological criteria: SynNeurGe (Synuclein-Neurodegeneration-Genetics) research diagnostic criteria for biological classification of PD (37) and NSD-ISS (Neuronal Synuclein Disease Integrated Staging System) (38). Both criteria aim to define PD (or NSD) based on biomarkers, with pathological α-syn detection as one of the cornerstones. However, in spite of great enthusiasm in the evolving field of α-syn biomarkers, especially α-syn seed amplification assays (SAA), α-syn positivity in control groups reached up to 5–10%—which is comparable to current MDS pPD research criteria specificity. Long-term data to confirm whether α-syn-positive individuals will develop clinically manifest synucleinopathies are therefore necessary. Moreover, despite unquestionable and exciting progress in the α-syn-SAA field, the technique had limited ability to confidently distinguish between pPD, pDLB, and pMSA (39, 40), although recent refinements and focus on amplification patterns, levels of fluorescence or aggregation kinetics showed promising results in differentiating PD and MSA in brain and CFS samples (41, 42). Thus, longitudinal studies are needed to determine whether biomarker-based diagnostics will complement or eventually replace current pPD criteria (4, 43).

### 4.6 Limitations and strengths

Several limitations have to be acknowledged. First, generalizability of our findings may seem limited by the recruitment strategy, which could overrepresent gastrointestinal symptoms; however, not all participants reported such complaints, and colonoscopy indications ranged from symptomatic to routine preventive screening. Also, inclusion criteria requiring at least one prodromal or risk marker (intended for an enriched cohort) introduced selection bias compared to the general population. Nevertheless, the relatively low detected pPD prevalence and the fact that vast majority of participants were ultimately classified as not meeting pPD criteria suggest that the cohort’s overall profile more closely resembles the general aging population than high-risk groups such as iRBD cohorts, supporting broader relevance of our findings.

Another challenge was relatively high attrition (45.6% overall), but dropout modeling indicated that missingness was not significantly associated with baseline pPD probability or clinical markers, suggesting limited impact on the longitudinal results. The sample size—particularly within the phenoconverter subgroup—was limited, reducing statistical power for some exploratory analyses. To minimize false negatives, no formal adjustments for multiple comparisons were applied, but all findings are presented transparently and interpreted cautiously.

As for the diagnostic approach, differences in study protocols between baseline and FU could affect longitudinal consistency. The absence of key high-specificity biomarkers—such as PSG-confirmed iRBD, DaTscan, and genetic testing—in the full cohort may have affected diagnostic accuracy, though this reflects common logistical and ethical challenges in population-based studies. Motor assessments were performed by multiple raters over a prolonged period, most not movement disorder specialists; despite standardized MDS-UPDRS training, inter-rater variability remains a potential limitation.

Given current efforts to integrate biomarkers into a biological definition of PD, correlating clinical profiles and MDS criteria-based pPD probability scores with biomarker-based SynNeurGe classification/NSD-ISS staging would be valuable but was not possible since data collection preceded these biological criteria. Since FU, we have collected samples for additional biomarker exploration—including genetic profiling, α-syn detection, multiomics or microbiome analysis—but these ongoing analyses were unavailable for this manuscript. Thus, we retained a clinically focused scope aligned with the original intent of evaluating MDS pPD criteria’s performance primarily using clinical markers. Future biomarker analyses will enable more robust, biologically enriched modeling of prodromal PD trajectories.

Regarding strengths, this study features a five-year longitudinal design in a real-world, primarily non–iRBD-enriched cohort with diverse prodromal profiles, making it more representative of the general aging population than many high-risk cohorts. Detailed phenotyping was performed at both timepoints using the MDS pPD criteria, including retrospective reclassification to harmonize baseline data with updated definitions. Methodological rigor was ensured through dropout modeling, multiple imputation, and use of both categorical and continuous probability scores. Importantly, the study offers transparent reporting and highlights key challenges in applying the MDS criteria in lower-risk populations, supporting their refinement for broader clinical and research use.

## 5 Conclusion

At first glance, dealing with the original and updated MDS pPD research criteria in such details may seem redundant in the upcoming era of biomarker-based PD diagnosis. However, as the emerging diagnostic options require access to sophisticated genetic testing, nuclear medicine techniques, and most importantly, α-syn SAA/immunohistochemical confirmation (with techniques still evolving and not standardized sufficiently to be used in a routine clinical practice), this approach can be hardly implemented in a common clinical setting and particularly in geographical regions with limited access even to a standard professional healthcare in the area of movement disorders. Thus, both MDS criteria (clinical for PD and research for pPD), less biomarker-based and more clinically-oriented, stay highly relevant and recommended (43).

In our cohort, tracking the dynamics of patients’ clinical profiles over time demonstrated consistency in pPD probability as detected by the MDS criteria. However, their time-consuming and logistically demanding nature, combined with relatively low baseline sensitivity in low-risk populations (increasing sufficiently only shortly before phenoconversion) limit their utility for population-wide screening. Additionally, their specificity, while generally high, allows for the detection of atypical parkinsonian syndromes, likely even beyond synucleinopathies.

Moving forward, integrating clinical criteria with biomarkers may enhance prognostic accuracy, but further longitudinal validation of (not only) α-syn biomarkers is necessary before they can fully replace current MDS pPD criteria.

## Data Availability

The raw data supporting the conclusions of this article will be made available by the authors, without undue reservation.
