# Supplementary material for: Tracking prodromal Parkinson’s disease: a five-year follow-up of the PARCAS cohort
Source: Front Neurol. 2025 Sep 12;16:1631165. doi: 10.3389/fneur.2025.1631165 (PMC12464032; doi:10.3389/fneur.2025.1631165)
Supplement: Supplementary file 1 [file Table_1.docx]

**Supplementary Table 1:**

**Diagnostic tools and likelihood ratio cut-offs for baseline and follow-up assessments of risk and prodromal markers based on the MDS pPD research criteria**

| **Markers** | **Baseline examination** | | | **Follow-up examination** | | |
| --- | --- | --- | --- | --- | --- | --- |
|  | **Diagnostic tools** | **Criteria for LRs** | | **Diagnostic tools** | **Criteria for LRs** | |
| **RISK FACTORS** | | | | | | |
| **Male sex** | Self-report questionnaire | | • **LR+**: male  • **LR-**: female | Self-report questionnaire | | • **LR+**: male  • **LR-**: female |
| **Pesticide exposure** | Self-report questionnaire: *exposure to pesticides* | | • **LR+**: yes (otherwise not applicable) | Self-report questionnaire:  *regular occupational exposure to pesticides or very frequent (>100 episodes) nonoccupational exposure* | | • **LR+**: yes (otherwise not applicable) |
| **Solvent  exposure** | Self-report questionnaire: *exposure to solvents* | | • **LR+**: yes (otherwise not applicable) | Self-report questionnaire:  *occupational exposure to solvents* | | • **LR+**: yes (otherwise not applicable) |
| **Non-use  of caffeine** | Self-report questionnaire:  *reported regular coffee consumption* | | • **LR+**: no  • **LR-**: yes | Self-report questionnaire:  *reported ≥ 3 cups of coffee or ≥ 6 cups of tea per week* | | • **LR+**: no  • **LR-**: yes |
| **Smoking status** | Self-report questionnaire:  *smoking status* | | • **LR+:** never smoker • **LR-** (stratified): current/former smoker | Self-report questionnaire:  *smoking status* | | • **LR+:** never smoker  • **LR- (stratified)**: current/former smoker (minimum 1 pack-year) |
| **Positive family history of PD** | Self-report questionnaire:  *any family relative with PD* | | • **LR+:** yes  (otherwise not applicable) | Self-report questionnaire:  *any first-degree relative with PD* | | • **LR+:** yes  (otherwise not applicable) |
| **Substantia nigra hyperechogenicity** | Transcranial sonography: *SN area measurement* | | • **LR+:** ≥ 0.25 cm^2^  • **LR=1:**  ≥ 0.20 and < 0.25 cm^2^  • **LR-:** < 0.20 cm^2^ | Transcranial sonography: *SN area measurement* | | • **LR+:** ≥ 0.25 cm^2^  • **LR=1:**  ≥ 0.20 and < 0.25 cm^2^  • **LR-:** < 0.20 cm^2^ |
| **DM type 2** | Not assessed | | - | Self-report questionnaire:  *history of DM type 2* | | • **LR+:** yes  • **LR-:** no |
| **Physical inactivity** | Not assessed | | - | Self-report questionnaire:  *reported < 1 hour per week of activity causing increased*  *respiratory or heart rate or sweating* | | • **LR+:** yes  • **LR-:** no |
| **Low plasma urate  (only in men)** | Not assessed | | - | Laboratory assessment:  *plasma urate concentration* | | • **LR+:**  < 297.4 μmol/l • **LR=1:**  297.4–333.09 μmol/l  • **LR-:**  > 333.09 μmol/l |
| **PRODROMAL MARKERS** | | | | | | |
| **RBD** | RBDSQ | | • **LR+**: ≥ 5 p.  • **LR-:** < 5 p. | RBDSQ | | • **LR+**: ≥ 5 p.  • **LR-:** < 5 p. |
|  | PSG  (subset of cohort) | | • **LR+**: positive  • **LR-:** negative | PSG  (subset of cohort) | | • **LR+**: positive  • **LR-:** negative |
| **Abnormal dopamine transporter imaging  (DaT-SPECT)** | Not assessed | | - | DaT-SPECT imaging | | • **LR+:**  abnormal finding  • **LR=1:**  nonspecific changes  • **LR-:**  normal finding |
| **Subthreshold parkinsonism** | MDS-UPDRS part III *(excluding postural and action tremor)* | | • **LR+:** > 6 p.  • **LR-:** ≤ 6 p. | MDS-UPDRS part III *(excluding postural and action tremor)* | | • **LR+:** > 6 p.  • **LR-:** ≤ 6 p. |
| **Hyposmia** | SS-12 | | • **LR+:** < 9 p.  • **LR-:** ≥ 9 p. | SS-16 | | • **LR+:**  ≤10^th^ percentile based on age and sex  • **LR-:** >10^th^ percentile |
| **Constipation** | Self-report questionnaire: *≤ 3 bowel movements per week* | | • **LR+:** yes  • **LR-:** no | MDS-UPDRS item 1.11: *constipation problems* | | • **LR+:** ≥ 2 p.  • **LR=1:** 1 p.  • **LR-:** 0 p. |
| **Excessive daytime somnolence** | MDS-UPDRS item 1.8: *daytime sleepiness* | | • **LR+:** ≥ 2 p.  • **LR=1:** 1 p.  • **LR-:** 0 p. | ESS | | • **LR+:** > 10 p.  • **LR-:**  ≤ 10 p. |
| **Symptomatic hypotension** | MDS-UPDRS item 1.12: *light headedness on standing* | | • **LR+:** ≥ 2 p.  • **LR=1:** 1 p.  • **LR-:** 0 p. | Orthostatic test | | • **LR+:**  – i-sBP ≥ 160 mmHg:  ≥ 30 mmHg reduction;  – i-sBP 120 –140 mmHg: ≥ 20 mmHg reduction; – i-sBP < 120 mmHg: ≥ 15 mmHg reduction or any reduction that ends in BP < 90 mmHg  • **LR=1:**  between LR+ and LR-  • **LR-:**  any i-sBP reduction < 10 mmHg |
| **Erectile dysfunction (only in men)** | Self-report questionnaire and structured interview: *presence of severe erectile dysfunction requiring treatment* | | • **LR+:** yes  • **LR-:** no | NMSS, item 26 (severity): *sexual function* | | • **LR+:** ≥ 2 p.  • **LR=1:** 1 p.  • **LR-:** 0 p. |
| **Urinary dysfunction** | MDS-UPDRS item 1.10: *urinary problems* | | • **LR+:** ≥ 2 p.  • **LR=1:** 1 p.  • **LR-:** 0 p. | MDS-UPDRS item 1.10:  *urinary problems* | | • **LR+:** ≥ 2 p.  • **LR=1:** 1 p.  • **LR-:** 0 p. |
| **Depression** | Self-report questionnaire and structured interview: *history of depression, non-induced by serious life events* | | • **LR+:** yes  • **LR-:** no | BDI-II, PAS | | • **LR+:** BDI-II ≥ 14 p. or previously treated depression  • **LR=1:** BDI-II < 14 p., PAS ≥ 14 p.  • **LR-:** BDI-II < 14 p., PAS < 14 p. |
| **Global cognitive deficit** | Not assessed | | - | MoCA | | • **LR+:** ≤ 25 p.  • **LR-:** > 25 p. |

Abbreviations: BDI-II: Beck Depression Inventory-II; BP: blood pressure; DaT: dopamine transporter; DM: diabetes mellitus; ESS: Epworth Sleepiness Scale; i-sBP: initial systolic BP; LR: likelihood ratio; LR+: positive LR; LR-: negative LR; LR=1: LR equals 1 (neutral contribution); MDS: International Parkinson and Movement Disorders Society; MDS-UPDRS: MDS-Unified Parkinson’s Disease Rating Scale; MoCA: Montreal Cognitive Assessment; NMSS: Non-Motor Symptoms Scale for Parkinson’s Disease; p: points; PAS: Parkinson Anxiety Scale; PD: Parkinson´s disease; PSG: polysomnography; RBD: REM Sleep Behaviour Disorder; RBDSQ: RBD Screening Questionnaire; SN: substantia nigra; SPECT: single-photon emission computed tomography; SS-12: 12-item Sniffin´ Sticks Identification test; SS-16: 16-item Sniffin´ Sticks Identification test.
