# Supplementary material for: Tracking prodromal Parkinson’s disease: a five-year follow-up of the PARCAS cohort
Source: Front Neurol. 2025 Sep 12;16:1631165. doi: 10.3389/fneur.2025.1631165 (PMC12464032; doi:10.3389/fneur.2025.1631165)
Supplement: Supplementary file 2 [file Table_2.docx]

**Supplementary Table 2:
Binary logistic regression model of dropout predictors across 10 imputed datasets**

| **Variable** | **B** | **S.E.** | **p** | **OR [95% CI]** | **Fraction Missing Information** | **Relative Increase Variance** | **Relative Efficiency** |
| --- | --- | --- | --- | --- | --- | --- | --- |
| **Sex** (females) | -0.162 | 0.407 | 0.690 | 0.850 [0.383, 1.889] | 0.030 | 0.031 | 0.997 |
| **Age** (years) | 0.007 | 0.027 | 0.798 | 1.007 [0.955, 1.062] | 0.111 | 0.122 | 0.989 |
| **Education** (years) | -0.125 | 0.060 | 0.037* | 0.882 [0.784 – 0.993] | 0.076 | 0.081 | 0.992 |
| **Exposure to pesticides** | 0.016 | 0.493 | 0.974 | 1.016 [0.387, 2.669] | 0.206 | 0.248 | 0.980 |
| **Exposure to solvents** | 0.193 | 0.803 | 0.810 | 1.213 [0.251, 5.859] | 0.363 | 0.530 | 0.965 |
| **Smoking** **status** -former  -current | -0.310  -0.425 | 0.479  0.663 | 0.517  0.522 | 0.733 [0.287, 1.876]  0.653 [0.178, 2.398] | 0.091  0.215 | 0.098  0.262 | 0.991  0.979 |
| **Nonuse of** **caffeine** | 0.297 | 0.443 | 0.503 | 1.346 [0.565, 3.205] | 0.156 | 0.178 | 0.985 |
| **Family history of PD** | -0.103 | 0.520 | 0.843 | 0.902 [0.326, 2.497] | 0.041 | 0.043 | 0.996 |
| **Echogenicity of SN:**  -borderline  -hyperechogenic | 0.565  0.605 | 0.722  0.695 | 0.437  0.386 | 1.760 [0.426, 7.259]  1.831 [0.472, 7.106] | 0.405  0.291 | 0.629  0.387 | 0.961  0.972 |
| **RBD** (based on RBDSQ) | 0.469 | 0.638 | 0.463 | 1.598 [0.458, 5.578] | 0.231 | 0.286 | 0.977 |
| **Subthreshold parkinsonism** | -0.687 | 0.524 | 0.190 | 0.503 [0.180, 1.401] | 0.038 | 0.039 | 0.996 |
| **Hyposmia** | 0.144 | 0.521 | 0.783 | 1.154 [0.416, 3.209] | 0.066 | 0.069 | 0.993 |
| **Constipation** | -0.146 | 0.419 | 0.727 | 0.864 [0.377, 1.981] | 0.046 | 0.047 | 0.995 |
| **Excessive daytime somnolence:** -borderline  -positive | 0.271  0.226 | 0.516  0.546 | 0.600  0.679 | 1.311 [0.475, 3.618]  1.253 [0.430, 3.649] | 0.016  0.058 | 0.017  0.061 | 0.998  0.994 |
| **Orthostatic hypotension:**  -borderline -positive | 0.780  1.240 | 0.510  0.880 | 0.127  0.160 | 2.180 [0.801, 5.943]  3.457 [0.635, 18.823] | 0.096  0.217 | 0.104  0.266 | 0.990  0.979 |
| **Urinary dysfunction:** -borderline  -positive | -0.558  -0.543 | 0.504  0.618 | 0.268  0.380 | 0.572 [0.213, 1.534]  0.581 [0.172, 1.963] | 0.065  0.082 | 0.068  0.088 | 0.994  0.992 |
| **Depression** | -0.294 | 0.566 | 0.603 | 0.745 [0.246, 2.259] | 0.149 | 0.169 | 0.985 |
| **pPD probability score** (%) ** | 0.003 | 0.014 | 0.816 | 1.003 [0.976, 1.031] | 0.163 | 0.189 | 0.984 |

*: p < 0.05
**: based on the updated MDS pPD criteria

Abbreviations: B: regression coefficient; OR [95% CI]: odds ratio with 95% confidence interval calculated from B; PD: Parkinson´s disease; pPD: prodromal PD; RBD: REM Sleep Behavior Disorder; RBDSQ: RBD Screening Questionnaire; S.E.: standard error of B; p.: p-value (statistical significance); SN: substantia nigra
