# Supplementary material for: Tracking prodromal Parkinson’s disease: a five-year follow-up of the PARCAS cohort
Source: Front Neurol. 2025 Sep 12;16:1631165. doi: 10.3389/fneur.2025.1631165 (PMC12464032; doi:10.3389/fneur.2025.1631165)
Supplement: Supplementary file 3 [file Table_3.docx]

**Supplementary Table 3:**

**Wilcoxon signed-ranks test assessing changes in prodromal Parkinson’s disease probability scores (based on the updated MDS pPD research criteria) from baseline to follow-up in the complete-case sample and across 10 imputed datasets**

| **Datasets** | **n** | **Wilcoxon signed-ranks test** | | | | | **Test statistics** | | |
| --- | --- | --- | --- | --- | --- | --- | --- | --- | --- |
|  |  | **Negative ranks ^a^** | | **Positive ranks ^b^** | | **Ties ^c^** | **Z** | **p** | **r** |
|  |  | **n** | **Sum of ranks** | **n** | **Sum of ranks** | **N** |  |  |  |
| Original | 86 | 48 | 2161.00 | 37 | 1494.00 | 1 | -1.461 ^d^ | 0.144 | 0.158 |
| 1 | 159 | 68 | 5631.00 | 90 | 6930.00 | 1 | -1.128 ^e^ | 0.260 | 0.090 |
| 2 | 159 | 66 | 5506.00 | 92 | 7055.00 | 1 | -1.345 ^e^ | 0.179 | 0.107 |
| 3 | 159 | 68 | 5564.00 | 90 | 6997.00 | 1 | -1.244 ^e^ | 0.214 | 0.099 |
| 4 | 159 | 70 | 5708.00 | 88 | 6853.00 | 1 | -0.994 ^e^ | 0.320 | 0.079 |
| 5 | 159 | 70 | 5692.00 | 88 | 6869.00 | 1 | -1.022 ^e^ | 0.307 | 0.081 |
| 6 | 159 | 71 | 5677.00 | 87 | 6884.00 | 1 | -1.048 ^e^ | 0.295 | 0.083 |
| 7 | 159 | 68 | 5521.00 | 90 | 7040.00 | 1 | -1.318 ^e^ | 0.187 | 0.105 |
| 8 | 159 | 66 | 5489.00 | 92 | 7072.00 | 1 | -1.374 ^e^ | 0.169 | 0.109 |
| 9 | 159 | 69 | 5639.00 | 89 | 6922.00 | 1 | -1.114 ^e^ | 0.265 | 0.089 |
| 10 | 159 | 68 | 5587.00 | 90 | 6974.00 | 1 | -1.204 ^e^ | 0.229 | 0.096 |
| Pooled results / average | 159 | 68.4 | 5601.40 | 89.6 | 6959.60 | 1 | -1.179 ^e^ | 0.243 | 0.096 |

^a^: FU pPD probability < baseline pPD probability
^b^: FU pPD probability > baseline pPD probability
^c^: FU pPD probability = baseline pPD probability
^d^: based on positive ranks
^e^: based on negative ranks

Abbreviations: n: number of participants in the dataset; p: p-value (statistical significance); pPD: prodromal Parkinson´s disease; r: effect size (calculated as *r = Z / √N*, where *N* is the number of non-tied pairs – for original data: N = 85, for imputed datasets: N = 158); Z: Wilcoxon test statistic (standardized).
