# Supplementary material for: Tracking prodromal Parkinson’s disease: a five-year follow-up of the PARCAS cohort
Source: Front Neurol. 2025 Sep 12;16:1631165. doi: 10.3389/fneur.2025.1631165 (PMC12464032; doi:10.3389/fneur.2025.1631165)
Supplement: Supplementary file 4 [file Table_4.docx]

**Supplementary Table 4:**

**Marginal homogeneity test (Stuart-Maxwell test) assessing changes in prodromal Parkinson’s disease categorical status (based on the updated MDS pPD research criteria) from baseline to follow-up in the complete-case sample and across 10 imputed datasets**

| **Datasets** | **n** | **Marginal homogeneity test^a^** | | | | **MH Statistics** | | | | | |
| --- | --- | --- | --- | --- | --- | --- | --- | --- | --- | --- | --- |
|  |  | **Off-diagonal cases** | | | **No change (ties)** ^d^ | **Observed MH** | **Mean MH** | **SD MH** | **Z** | **p** | **r** |
|  |  | **Total** | **↓ shifts** ^b^ | **↑ shifts** ^c^ |  |  |  |  |  |  |  |
| **Original** | 86 | 9 | 4 | 5 | 77 | 8.0 | 8.5 | 1.936 | -0.258 | 0.796 | 0.028 |
| 1 | 159 | 19 | 14 | 5 | 140 | 25.0 | 17.0 | 3.391 | 2.359 | 0.018* | 0.187 |
| 2 | 159 | 19 | 14 | 5 | 140 | 25.0 | 17.0 | 3.391 | 2.359 | 0.018* | 0.187 |
| 3 | 159 | 19 | 14 | 5 | 140 | 25.0 | 17.0 | 3.391 | 2.359 | 0.018* | 0.187 |
| 4 | 159 | 19 | 14 | 5 | 140 | 25.0 | 17.0 | 3.391 | 2.359 | 0.018* | 0.187 |
| 5 | 159 | 19 | 14 | 5 | 140 | 25.0 | 17.0 | 3.391 | 2.359 | 0.018* | 0.187 |
| 6 | 159 | 19 | 14 | 5 | 140 | 25.0 | 17.0 | 3.391 | 2.359 | 0.018* | 0.187 |
| 7 | 159 | 19 | 14 | 5 | 140 | 25.0 | 17.0 | 3.391 | 2.359 | 0.018* | 0.187 |
| 8 | 159 | 19 | 14 | 5 | 140 | 25.0 | 17.0 | 3.391 | 2.359 | 0.018* | 0.187 |
| 9 | 159 | 19 | 14 | 5 | 140 | 25.0 | 17.0 | 3.391 | 2.359 | 0.018* | 0.187 |
| 10 | 159 | 19 | 14 | 5 | 140 | 25.0 | 17.0 | 3.391 | 2.359 | 0.018* | 0.187 |
| **Pooled results / average** | 159 | 19 | 14 | 5 | 140 | 25.0 | 17.0 | 3.391 | 2.359 | 0.018* | 0.187 |

^a^: 3 distinct values: no pPD (pPD probability < 50%), possible pPD (50-79.99%), probable pPD (≥80%)
^b^: FU pPD category < baseline pPD category
^c^: FU pPD category > baseline pPD category
^d^: FU pPD category = baseline pPD category
*: p < 0.05

Abbreviations: MH: marginal homogeneity; n: number of participants in the dataset; p: p-value (statistical significance); pPD: prodromal Parkinson´s disease; r: effect size (calculated as *r = Z / √N*, where *N* is the number of total paired observations); SD: standard deviation of MH statistic; Z: standardized MH statistic.
