# Supplementary material for: Tracking prodromal Parkinson’s disease: a five-year follow-up of the PARCAS cohort
Source: Front Neurol. 2025 Sep 12;16:1631165. doi: 10.3389/fneur.2025.1631165 (PMC12464032; doi:10.3389/fneur.2025.1631165)
Supplement: Supplementary file 5 [file Data_Sheet_1.docx]

Supplementary Materials and Methods

***Diagnostic tools for calculating the probability of prodromal Parkinson's disease based on the MDS research criteria***

Since the baseline examinations were conducted before the publication of the updated MDS pPD research criteria in 2019 (1), only risk factors and prodromal markers from the original MDS pPD criteria were included (2). At follow-up (FU), all risk and prodromal markers from the updated MDS criteria were assessed, except for genetic testing; DaTscan was performed in a selected subset of high-risk patients. In addition, the FU protocol was modified and expanded as described previously (3,4) to align with our prospective Parkinson’s Disease BIOMarker (PDBIOM) study which evaluates subjects with idiopathic REM-sleep behavior disorder (iRBD).

Most **RISK FACTORS** (**male sex, regular pesticide/solvent exposure, nonuse of caffeine, smoking status, positive family history of PD** at baseline and FU, and **physical inactivity, and type 2 diabetes mellitus** at FU) were assessed using a self-report questionnaire. **Hyperechogenicity of the substantia nigra** (SN) was evaluated by transcranial sonography with a 3-1MHz sector array transducer probe (Phillips HD11XE, Phillips Ultrasound, Bothwell, WA, USA), adopting the previously published protocol (5). Patients were evaluated by a single experienced examiner (NL). Positive likelihood ratio (LR+) was applied in case of the area of the SN of ≥ 0.25 cm^2^, LR=1 in case of a borderline result (0,2-0.24 cm^2^), and negative LR (LR-) in case of < 0.2 cm^2^. Males were screened for **low plasma urate levels** at FU (LR+ was applied for low values < 297.4 μmol/l, LR=1 for borderline levels of 297.4-333.09 μmol/l, and LR- for levels > 333.09 μmol/l).

As for the **PRODROMAL MARKERS**, standardized scales and clinical tests were used. The presence of **RBD** was determined using the RBD-Screening Questionnaire (RBDSQ) in most cases (LR+ for ≥ 5/13 points (p.), LR- for < 5 p.); full polysomnography was performed in a subset of patients with high RBDSQ scores willing to undergo more detailed examination. Due to economic and ethical considerations, **dopaminergic SPECT (**single-photon emission computed tomography; **DaTscan)** was offered only to a subset of high-risk patients meeting probable pPD status based on a comprehensive evaluation of the MDS criteria. LR+ was applied in case of a clearly abnormal finding; LR- for normal finding, and LR=1 in case of some abnormal findings not typical for PD (e.g. mild bilateral reduction of the presynaptic dopamine transporter). **Subthreshold parkinsonism** was assessed by the MDS-Unified Parkinson’s Disease Rating Scale (MDS-UPDRS) part III (6), excluding postural and action tremor (LR+ for > 6 p., LR- for ≤ 6 p.). **Hyposmia** was evaluated using the 12-item Sniffin´ Sticks Identification test (SS-12), culturally adapted for the Slovak population, at baseline (LR+ for < 9/12 p., LR- for ≥ 9 p.), while at FU, a more comprehensive 16-item version of the test (SS-16) was used (7) (for LR+, hyposmia was defined as scoring <10^th^ percentile, adjusted for age and sex, otherwise LR- was applied). **Constipation** was assessed as self-reported ≤ 3 bowel movements per week at baseline; and at FU, MDS-UPDRS item 1.11 was used (6) (LR+ for ≥ 2/4 p., LR=1 for a score of 1 p., and LR- for 0 p.). **Excessive daytime sleepiness** was evaluated using MDS-UPDRS item 1.8 (6) at baseline (LR+ for ≥ 2/4 p., LR=1 for 1 p., and LR- for 0 p.) and the Epworth Sleepiness Scale (ESS) (8) at FU (LR+ for > 10 p., and LR- for ≤ 10 p.). Presence of **symptomatic orthostatic hypotension** was determined by MDS-UPDRS item 1.12 (6) at baseline (LR+ for ≥ 2/4 p., LR=1 for 1 p., and LR- for 0 p.), and an orthostatic test at FU (9) (measurement of blood pressure (BP) after 3 minutes supine and subsequently after 3 minutes in a standing position; LR+ was applied based on a significant supine-to-standing BP reduction, graded as follows: ≥ 30 mmHg reduction for initial systolic BP (sBP) ≥ 160 mmHg; ≥ 20 mmHg reduction for sBP 120–140 mmHg; and ≥ 15 mmHg or any reduction that ends in BP < 90 mmHg for initial sBP < 120 mmHg; LR- was used in the case of sBP reduction < 10 mmHg; otherwise LR=1 was used). As we could not eliminate alternative causes of OH (dehydration, cardiac disease, autonomic neuropathy, medication, etc.) required for OH to be considered as neurogenic, the more conservative LR+ was used when calculating based on the 2019 pPD criteria. **Severe erectile dysfunction** was evaluated using a self-reported questionnaire and structured interview at baseline, and the Non-Motor Symptoms Scale for Parkinson’s Disease (NMSS), item 26 (10) at FU (LR+ for score ≥ 2/3 p. in severity; LR=1 for 1 p., and LR- for 0 p.). **Urinary dysfunction** was assessed using MDS-UPDRS item 1.10 (6) (LR+ for ≥ 2/4 p., LR=1 for 1 p., and LR- for 0 p.) both at baseline and FU. **Depression** was screened by a self-report questionnaire and structured interview at baseline, and the Beck Depression Inventory-II (BDI-II) (11) at FU (LR+ for ≥ 14 p. on the BDI-II or a history of treated depression, LR=1 if the BDI-II score was < 14 p., but comorbid anxiety was present (defined as a score ≥ 14 p. in the Parkinson Anxiety Scale (PAS) (12)), and LR- for scores < 14 p. on both the BDI-II and PAS). Last, **global cognitive deficit** was assessed at FU using the Montreal Cognitive Assessment (MoCA) (13); (LR+ for ≤ 25/30 p., LR- for LR- for scores > 25 p.). When any of the values were missing, LR 1 was applied.

A detailed summary of the diagnostic protocols, including diagnostic tools and cut-off criteria for LR assignment, is provided in Supplementary Table 1 for a clear and visual reference.

**References**

1. Heinzel S, Berg D, Gasser T, Chen H, Yao C, Postuma RB, MDS Task Force on the Definition of Parkinson’s Disease. Update of the MDS research criteria for prodromal Parkinson’s disease. *Mov Disord* (2019) 34:1464–1470. doi: 10.1002/mds.27802

2. Berg D, Postuma RB, Adler CH, Bloem BR, Chan P, Dubois B, Gasser T, Goetz CG, Halliday G, Joseph L, et al. MDS research criteria for prodromal Parkinson’s disease. *Mov Disord* (2015) 30:1600–1611. doi: 10.1002/mds.26431

3. Skorvanek M, Ladomirjakova Z, Han V, Lesko N, Feketeova E, Jarcuskova D, Repkova B, Spisak P, Urbancikova Z, Vargova A, et al. Prevalence of Prodromal Parkinson’s Disease as Defined by MDS Research Criteria among Elderly Patients Undergoing Colonoscopy. *J Parkinsons Dis* (2017) 7:481–489. doi: 10.3233/JPD-161036

4. Kulcsarova K, Ventosa JR, Feketeova E, Maretta M, Lesko N, Benca M, Han V, Gombosova L, Baloghova J, Slavkovska M, et al. Comparison in detection of prodromal Parkinson’s disease patients using original and updated MDS research criteria in two independent cohorts. *Parkinsonism Relat Disord* (2021) 87:48–55. doi: 10.1016/j.parkreldis.2021.04.028

5. Walter U, Behnke S, Eyding J, Niehaus L, Postert T, Seidel G, Berg D. Transcranial brain parenchyma sonography in movement disorders: state of the art. *Ultrasound Med Biol* (2007) 33:15–25. doi: 10.1016/j.ultrasmedbio.2006.07.021

6. Goetz CG, Tilley BC, Shaftman SR, Stebbins GT, Fahn S, Martinez-Martin P, Poewe W, Sampaio C, Stern MB, Dodel R, et al. Movement Disorder Society-sponsored revision of the Unified Parkinson’s Disease Rating Scale (MDS-UPDRS): scale presentation and clinimetric testing results: MDS-UPDRS: Clinimetric Assessment. *Mov Disord* (2008) 23:2129–2170. doi: 10.1002/mds.22340

7. Rumeau C, Nguyen DT, Jankowski R. How to assess olfactory performance with the Sniffin’ Sticks test ®. *Eur Ann Otorhinolaryngol Head Neck Dis* (2016) 133:203–206. doi: 10.1016/j.anorl.2015.08.004

8. Johns MW. A new method for measuring daytime sleepiness: The Epworth sleepiness scale. *Sleep* (1991) 14:540–545. doi: 10.1093/sleep/14.6.540

9. Tzur I, Izhakian S, Gorelik O. Orthostatic hypotension: definition, classification and evaluation. *Blood Press* (2019) 28:146–156. doi: 10.1080/08037051.2019.1604067

10. Chaudhuri KR, Martinez-Martin P, Brown RG, Sethi K, Stocchi F, Odin P, Ondo W, Abe K, Macphee G, Macmahon D, et al. The metric properties of a novel non-motor symptoms scale for Parkinson’s disease: Results from an international pilot study. *Mov Disord* (2007) 22:1901–1911. doi: 10.1002/mds.21596

11. Beck AT, Steer RA, Brown GK. *Manual for the Beck Depression Inventory-II, Psychological Corporation*. San Antonio. (1996).

12. Leentjens AFG, Dujardin K, Pontone GM, Starkstein SE, Weintraub D, Martinez-Martin P. The Parkinson Anxiety Scale (PAS): development and validation of a new anxiety scale: THE PARKINSON ANXIETY SCALE (PAS). *Mov Disord* (2014) 29:1035–1043. doi: 10.1002/mds.25919

13. Nasreddine ZS, Phillips NA, Bédirian V, Charbonneau S, Whitehead V, Collin I, Cummings JL, Chertkow H. The Montreal Cognitive Assessment, MoCA: A brief screening tool for mild cognitive impairment. *J Am Geriatr Soc* (2005) 53:695–699. doi: 10.1111/j.1532-5415.2005.53221.x
