# Supplementary material for: Tracking prodromal Parkinson’s disease: a five-year follow-up of the PARCAS cohort
Source: Front Neurol. 2025 Sep 12;16:1631165. doi: 10.3389/fneur.2025.1631165 (PMC12464032; doi:10.3389/fneur.2025.1631165)
Supplement: Supplementary file 6 [file Data_Sheet_2.docx]

Supplementary Results

1. ***Prodromal Parkinson's disease identification based on the original MDS pPD research criteria (baseline & follow-up)***

At baseline, 160 subjects with sufficient data for pPD probability calculation were included in the PARCAS cohort (64 males - 40%, 96 females - 60%, mean age 62.36±9.16 years). Original MDS pPD research criteria identified 9 possible (5.6%) and 9 probable (5.6%) pPD cases.

After approximately 5 years, 87 participants (54.4%) completed follow-up (35 males - 40.2%, 52 females - 59.8%, mean age 67.18±8.58 y., mean time to FU: 5.46±1.24 y.). This FU subgroup of the cohort included 8 possible (9.2%) and 2 probable pPD cases (2.3%) based on the original MDS criteria at baseline. At FU, one patient had already phenoconverted to manifest neurodegenerative disease and was excluded from further pPD assessment. For the remaining 86 subjects, the original MDS criteria identified 4 possible (4.7%) and 3 probable (3.5%) pPD cases (79 subjects - 91.9% - were still negative).

***2. Evolution of prodromal Parkinson's disease probability in individual participants based on the original MDS pPD research criteria (baseline vs. FU):***

According to the original MDS pPD criteria, 76 participants (88.4%) remained in the same pPD category as at baseline (75 still negative, 1 still possible pPD). 10 participants (11.6%) experienced a categorical shift – 6 individuals (7.0%) moved to a lower-risk category (2 from probable to possible pPD, and 4 from possible pPD to negative), while 4 (4.6%) moved to a higher-risk category (2 newly identified cases: 1 possible, 1 probable pPD; and 2 progressed from possible to probable pPD). Regarding changes in continuous pPD probability scores, 52 participants (60.5%) experienced a decrease, 33 (38.4%) an increase, and 1 (1.2%) showed no change over time.

To address potential attrition bias due to missing follow-up data (45.6% dropout), multiple imputation was performed using baseline age, sex, risk and prodromal markers, and baseline pPD probability (based on the 2015 criteria) as predictors. Implausible imputed values exceeding the 0–100% range were corrected accordingly (0,1% of imputed values). Follow-up pPD probability was imputed across 10 datasets.

The complete-case Wilcoxon signed-rank test revealed a statistically significant decrease in pPD probability from baseline to follow-up (Z = –2.756, p = 0.006, moderate estimated effect size: r = 0.299). In contrast, results from the imputed datasets consistently showed no significant change (average Z = –0.147, average p = 0.884, very small estimated effect size: r = 0.012), suggesting that the apparent decrease observed in the complete-case analysis may have been driven by attrition-related bias. These findings indicate that, after accounting for missing data, there is no robust evidence of a directional change in pPD probability over time.

Regarding categorical changes, the marginal homogeneity test (Stuart–Maxwell test) also showed no significant shift in pPD status categories in the complete-case analysis (Z = 0.277, p = 0.782, negligible estimated effect size: r = 0.030). However, in the imputed datasets, the test consistently indicated a statistically significant shift toward **lower** prodromal categories over time (average Z = 2.469, average p = 0.014, small estimated effect size: average r = 0.196). This seemingly paradoxical finding—given the relative stability in continuous scores—likely reflects small, non-directional fluctuations around diagnostic thresholds that lead to categorical reclassification. It underscores how even subtle changes in probability can have non-linear effects on classification, and how imputation may reveal attrition-masked patterns not apparent in the observed data alone.

pPD probability and category changes from baseline to FU in the complete-case sample are presented in Supplementary Fig. 1, for both the entire cohort and individual phenoconverters.


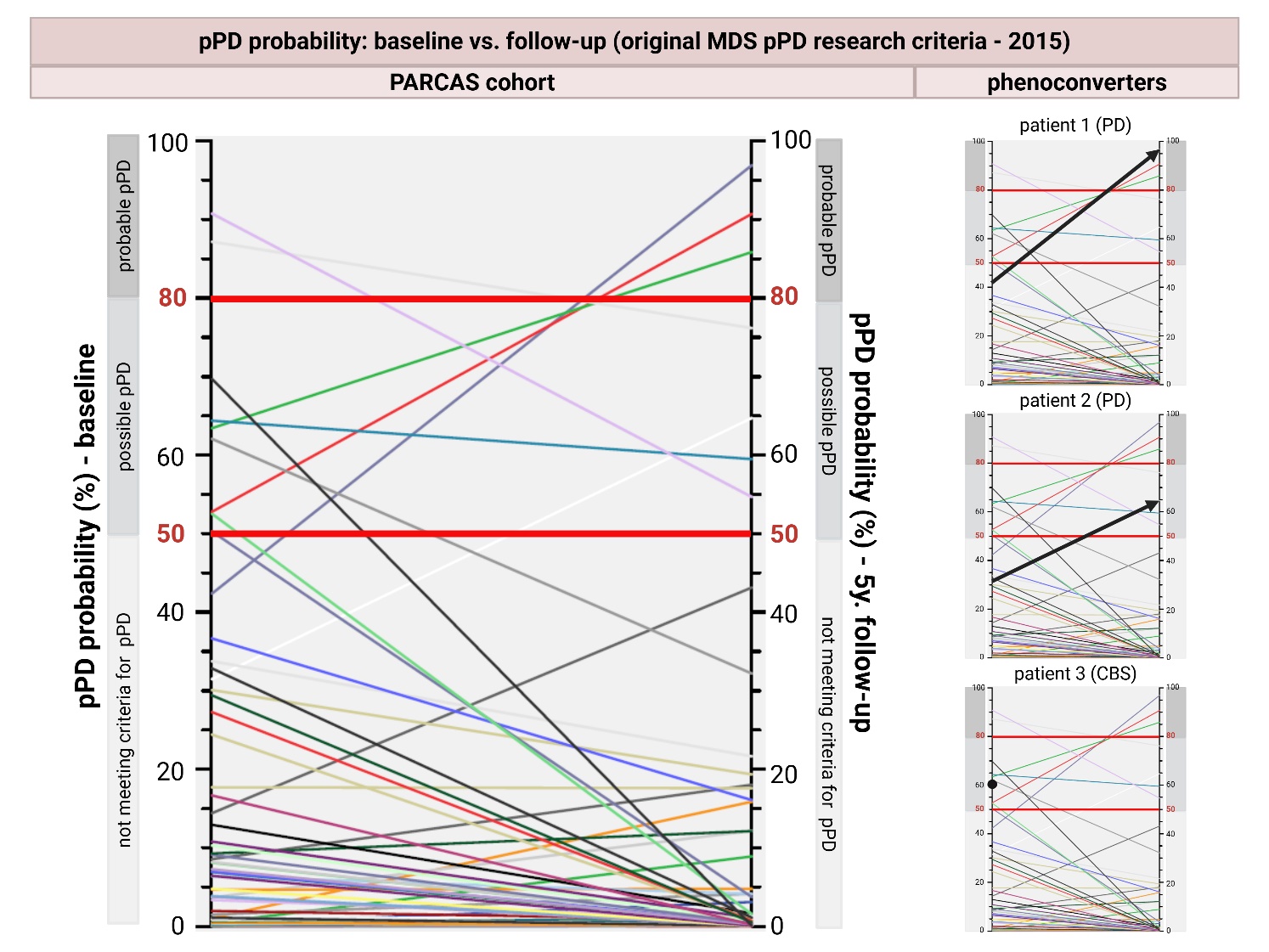


**Supplementary Figure 1: Evolution of prodromal Parkinson's disease probability (%) as defined by the original MDS research criteria - from baseline to follow-up after 5 years.**
Left side: summary graph for the entire PARCAS cohort; right side: individual phenoconverted patients highlighted; red lines - 50% and 80% probability cut-offs for possible and probable pPD, respectively; individual narrow color lines representing individual participants and their % pPD probability change from baseline to follow-up; individual bold black arrows/dot representing phenoconverters.
Abbreviations: CBS: corticobasal syndrome; pPD: prodromal Parkinson's disease; y: years.

**Supplementary Table 5:**

**Wilcoxon signed-ranks test assessing changes in prodromal Parkinson’s disease probability scores (based on the original MDS pPD research criteria) from baseline to follow-up in the complete-case sample and across 10 imputed datasets**

| **Datasets** | **n** | **Wilcoxon signed-ranks test** | | | | | **Test statistics** | | |
| --- | --- | --- | --- | --- | --- | --- | --- | --- | --- |
|  |  | **Negative ranks ^a^** | | **Positive ranks ^b^** | | **Ties ^c^** | **Z** | **p** | **r** |
|  |  | **n** | **Sum of ranks** | **n** | **Sum of ranks** | **N** |  |  |  |
| Original | 86 | 52 | 2456.50 | 33 | 1198.50 | 1 | -2.756 ^d^ | 0.006* | 0.299 |
| 1 | 159 | 77 | 6508.50 | 81 | 6052.50 | 1 | -0.396 ^e^ | 0.692 | 0.032 |
| 2 | 159 | 74 | 6316.50 | 84 | 6244.50 | 1 | -0.062 ^e^ | 0.950 | 0.005 |
| 3 | 159 | 74 | 6224.50 | 84 | 6336.50 | 1 | -0.097 ^e^ | 0.923 | 0.008 |
| 4 | 159 | 77 | 6481.50 | 81 | 6079.50 | 1 | -0.340 ^e^ | 0.727 | 0.027 |
| 5 | 159 | 73 | 6378.50 | 85 | 6182.50 | 1 | -0.170 ^e^ | 0.865 | 0.014 |
| 6 | 159 | 75 | 6267.50 | 83 | 6293.50 | 1 | -0.023 ^e^ | 0.982 | 0.002 |
| 7 | 159 | 73 | 6221.50 | 85 | 6339.50 | 1 | -0.102 ^e^ | 0.918 | 0.008 |
| 8 | 159 | 73 | 6220.50 | 85 | 6340.50 | 1 | -0.104 ^e^ | 0.917 | 0.008 |
| 9 | 159 | 74 | 6363.50 | 84 | 6197.50 | 1 | -0.144 ^e^ | 0.885 | 0.011 |
| 10 | 159 | 73 | 6297.50 | 85 | 6263.50 | 1 | -0.030 ^e^ | 0.976 | 0.002 |
| Pooled results / average | 159 | 74.3 | 6328.05 | 83.7 | 6233.00 | 1 | -0.147 | 0.884 | 0.012 |

^a^: FU pPD probability < baseline pPD probability
^b^: FU pPD probability > baseline pPD probability
^c^: FU pPD probability = baseline pPD probability
^d^: based on positive ranks
^e^: based on negative ranks
*: p < 0.05

Abbreviations: n: number of participants in the dataset; p: p-value (statistical significance); pPD: prodromal Parkinson´s disease; r: effect size (calculated as *r = Z / √N*, where *N* is the number of non-tied pairs – for original data: N = 85, for imputed datasets: N = 158); Z: Wilcoxon test statistic (standardized).

**Supplementary Table 6:**

**Marginal homogeneity test (Stuart-Maxwell test) assessing changes in prodromal Parkinson’s disease categorical status (based on the original MDS pPD research criteria) from baseline to follow-up in the complete-case sample and across 10 imputed datasets**

| **Datasets** | **n** | **Marginal homogeneity test ^a^** | | | | **MH Statistics** | | | | | |
| --- | --- | --- | --- | --- | --- | --- | --- | --- | --- | --- | --- |
|  |  | **Off-diagonal cases** | | | **No change (ties)** ^d^ | **Observed MH** | **Mean MH** | **SD MH** | **Z** | **p** | **r** |
|  |  | **Total** | **↓ shifts** ^b^ | **↑ shifts** ^c^ |  |  |  |  |  |  |  |
| **Original** | 86 | 10 | 6 | 4 | 76 | 10.0 | 9.5 | 1.803 | 0.277 | 0.782 | 0.030 |
| 1 | 159 | 18 | 14 | 4 | 141 | 25.0 | 17.0 | 3.240 | 2.469 | 0.014* | 0.196 |
| 2 | 159 | 18 | 14 | 4 | 141 | 25.0 | 17.0 | 3.240 | 2.469 | 0.014* | 0.196 |
| 3 | 159 | 18 | 14 | 4 | 141 | 25.0 | 17.0 | 3.240 | 2.469 | 0.014* | 0.196 |
| 4 | 159 | 18 | 14 | 4 | 141 | 25.0 | 17.0 | 3.240 | 2.469 | 0.014* | 0.196 |
| 5 | 159 | 18 | 14 | 4 | 141 | 25.0 | 17.0 | 3.240 | 2.469 | 0.014* | 0.196 |
| 6 | 159 | 18 | 14 | 4 | 141 | 25.0 | 17.0 | 3.240 | 2.469 | 0.014* | 0.196 |
| 7 | 159 | 18 | 14 | 4 | 141 | 25.0 | 17.0 | 3.240 | 2.469 | 0.014* | 0.196 |
| 8 | 159 | 18 | 14 | 4 | 141 | 25.0 | 17.0 | 3.240 | 2.469 | 0.014* | 0.196 |
| 9 | 159 | 18 | 14 | 4 | 141 | 25.0 | 17.0 | 3.240 | 2.469 | 0.014* | 0.196 |
| 10 | 159 | 18 | 14 | 4 | 141 | 25.0 | 17.0 | 3.240 | 2.469 | 0.014* | 0.196 |
| **Pooled results / average** | 159 | 18 | 14 | 4 | 141 | 25.0 | 17.0 | 3.240 | 2.469 | 0.014* | 0.196 |

^a^: 3 distinct values: no pPD (pPD probability < 50%), possible pPD (50-79.99%), probable pPD (≥80%)
^b^: FU pPD category < baseline pPD category
^c^: FU pPD category > baseline pPD category
^d^: FU pPD category = baseline pPD category
*: p < 0.05

Abbreviations: MH: marginal homogeneity; n: number of participants in the dataset; p: statistical significance; pPD: prodromal Parkinson´s disease; r: effect size (calculated as *r = Z / √N*, where *N* is the number of total paired observations); SD: standard deviation of MH statistic; Z: standardized MH statistic.
